# Hospital and Institutional News

**Published:** 1919-06-14

**Authors:** 


### HOSPITAL AND INSTITUTIONAL NEWS.

### THE LATE MR. GEORGE COOPER FRANKLIN.

By the death of Mr. G. C. Franklin Leicester Royal Infirmary loses its Senior Consulting Surgeon. Mr. Franklin was born in Leicester in 1846, and qualified M.R.C.S. in 1870 and L.R.C.P. in 1871, from St. Thomas's Hospital, London. He was admitted F.R.C.S. in 1873. He filled the posts of House Surgeon and Resident Accoucheur at St. Thomas's Hospital, and subsequently held office as R.M.O. at the Hospital for Diseases of the Chest. He was president of numerous societies in Leicester—Medical, Scientific and Musical—but the culminating honour was his election as President of the British Medical Association which met in Leicester in 1905. He was for five years Surgeon to Leicester Provident Dispensary, and in 1886 was appointed Surgeon to Leicester Royal Infirmary, which post he resigned in 1906, on reaching the age limit. In recognition of his services to the Infirmary he was, on April 25, 1906, entertained to dinner by the practitioners of the town and many prominent citizens. On leaving Leicester, Mr. Franklin made his home at Fareham, Hants, and on the outbreak of war was appointed Commandant and Medical Officer in charge of the British Red Cross Hospital at Hawkstone, in the Salisbury Command, which services were recognised in his appointment to M.B.E. On the closing of this Hospital in January last, Mr. Franklin undertook duties on the Medical Advisory Committee at Portsmouth.

### SANATORIA FOR SOLDIERS AND SAILORS.

The Inter-Departmental Committee on Tuberculosis amongst Discharged Soldiers and Sailors, comprising representatives from the Local Government Boards for England, Scotland, and Ireland, the National Health Insurance Commissions for England and Ireland, the Board of Agriculture, the Ministry of Labour and the Ministry of Pensions, together with Dr. Nathan Raw, M.P., Sir Owen Thomas, M.P., and Sir Kingsley Wood, M.P., and presided over by Sir Montague Barlow, has held nine meetings and has heard a large amount of evidence from Government Departments, local authorities, directors of sanatoria and of colonies for disabled men, and from soldiers' organisations, etc. The Committee have begun considering their report, and hope, soon after Parliament reassembles, to publish their recommendations, with a view to prompt steps being taken to secure adequate solution of this urgent problem and full provision of treatment for all tuberculous discharged soldiers and sailors.

### RECOGNITION OF HOME WAR SERVICE.

Battersea Borough Council have performed a gracious act in presenting to Dr. C. E. McDade an illuminated address recording the Council's appreciation of the services rendered by him as Acting Medical Officer of Health from March 1915 to April 1919, during the absence of Dr. G. Q. Lennane on active service. Their appreciation did not end with the address, for with it was handed to Dr. McDade a cheque for one hundred guineas. The position was a trying one, for not only had Dr. McDade to face a period of great tension, but to work with a greatly depleted staff. Happily, he was able to satisfy the most exacting; hence the Council's recognition of his services in a tangible form.

### PSYCHOTHERAPY IN THE MIDLANDS.

A psychotherapeutic clinic has been established in connection with the Birmingham and Midland Hospital for Nervous Diseases for those patients, more than half the total, where disorder is chiefly mental in origin. To carry out the postponed extension scheme a property within a few minutes' walk of the present hospital is now the subject of negotiations. In grounds of three acres the proposed building could accommodate thirty patients, but a special appeal for funds for its purchase and equipment will be required. It is interesting to note that the board favours the Scottish plan of placing a medical superintendent at the head of the institution, and has appointed Dr. Alfred Carver to fill this post. The financial year ended with a small balance in hand, and if Dr. Carver and his staff can raise sufficient money to enter the new premises free of debt, an important development will have been very successfully begun.

### THE GENERAL LYING-IN HOSPITAL TO-DAY.

One of the smaller hospitals of London which does not come before the public eye as much as it should is the General Lying-in Hospital in York Road, Lambeth. It has been in existence for more than 150 years. Nearly one thousand babies were born in the hospital last year. There are thirty-six beds, and 877 mothers were attended in their own homes. The hospital also possesses a baby clinic, whose advice is given to mothers on diet during three afternoons a week. For two years after birth the babies are under medical supervision, and visitors go to the homes of the children to see how the instructions given at the clinic have been understood and are being carried out. But babies, unlike other patients, cannot noise abroad their gratitude, nor can their mothers do much except to bring more cases to the hospital's doors. For this reason the General Lying-in Hospital labours under a peculiar difficulty, and the consequence is that it has only overdrafts to depend upon. It possesses however, in the person of Miss R. E. Whyte, a woman hospital secretary, and we look to women to provide their colleagues with new financial ideas. Hospitals are now describing their work by means of the cinematograph. There is also a certain palace near by with an old garden. Could not the wife of its occupier be persuaded to come to the rescue by a garden party on behalf of the hospital in her famous grounds?

### LONDON HOSPITAL SATURDAY FUND.

In our issue of May 3, we referred to the retirement of Mr. A. W. Davis as Secretary to the London Hospital Saturday Fund. At a recent meeting of the Board of Delegates it was decided that Mr. Davis should have six months' holiday granted him, as the Articles of Association prevent the granting of a pension, notwithstanding the fact that he had served the Fund for nearly twenty-five years. A resolution was also passed expressing appreciation of Mr. Davis's services. One hundred applications for the vacant post had been received. Five names were submitted to the Board. Mr. Philip A. Inman was elected. Although Mr. Inman had been associated with the work of the Fund for less than twelve months, he had shown by his energy and integrity that he thoroughly understands its works and aims. He gained his experience at Leeds, Birmingham, and Bath, and those who selected him have every confidence that he will prove a worthy successor to Mr. Davis.

### HOSPITAL AMALGAMATION IN BELFAST.

For a hospital to be able to live well within its income is a remarkable feat at present, but it has been accomplished by the Belfast Ophthalmic Hospital. Its future, however, is uncertain in two ways. The hospital will soon have to be enlarged, and at the time when this is being considered, the Benn Eye, Ear, and Throat Hospital is considering whether the amalgamation of the two hospitals can be arranged. The Ophthalmic Hospital is not opposed to the principle of amalgamation, but its board is bound by the trust not to dispose of the present premises, nor to apply the income from the estate of the late Lady Johnson to any purpose except that of an eye and ear hospital. The former point seems to be more difficult, unless the modern plan is favoured which keeps an out-patient department in the centre of a city and removes in-patients to new quarters on its outskirts. If the Benn Hospital is not tied to its site, there is the prospect that the scheme may be carried through.

### IRISH BANGOR.

Sir John Byers lately gave an address at a meeting of the Bangor Hospital, near Belfast, in which he sketched the historical growth of hospitals, especially in Ireland. After remarking that Ireland was the centre of European learning in the sixth and seventh centuries, he referred to such counties as Armagh, Bangor, Morilla, and Downpatrick, by the side of which hospitals began to flourish, since religion, learning, and the treatment of the sick once went hand in hand. About the same period leprosy spread in Northern Europe, and, in time, on the Irish coast and in England and Scotland 200 lazar-houses arose. Ireland also suffered from the prevalence of leprosy, and Sir John reminded his hearers that Leopardstown was once Leperstown. Bangor Hospital half-a-century ago was situated in the old street known as The Kennel. Then the cottage hospital was founded by Mrs. Ward, and in 1910 the present Bangor Hospital was opened.

### CAMBERWELL AND MATERNITY WELFARE.

Camberwell Borough Council has adopted a scheme which will practically cover the whole of its area as to maternity and child welfare. The Local Government Board were desirous that the Council should adopt a comprehensive scheme, but the Council was of opinion that the policy originally decided upon, to subsidise the existing voluntary organisations in the borough engaged in this work with the object of enabling them to extend and develop it, should be continued. It was desired that the whole of the borough should be covered as far as possible, and suggestions were made to three organisations to take over wards which were not covered. The organisations have agreed to do this, and the Council has accordingly increased the grants to them to the extent of £50 each. Altogether under the scheme the Council will contribute to six Maternity and Child Welfare Centres £605 a year, making in addition a special payment to the Ranyard Nurses for their attendance on cases.

### "MANY HOSPITALS TO BE CLOSED."

Speaking at a meeting of the Bristol Homœopathic Hospital, Dr. Burford handed over a cheque for £250 which the trustees of Neuilly Hospital in France had presented as a donation. Dr. Burford also referred to the Ministry of Health, which he thought would secure greater unification and would mean that a good many hospitals would have to be closed. This statement, if correctly reported, is rather curious, for the first question with which a Ministry of Health will have to deal is the shortage of hospital beds, and the object of unification is to make administration easier and to see that every available bed is at the disposal of waiting patients. What, then, does Dr. Burford mean?

### AN OLD HALL FOR PRESTON INFIRMARY.

Through Mr. Alexander Foster, a director, Messrs. George and R. Dewhurst, Ltd., cotton manufacturers, have presented Lostock Hall, for use as a convalescent home, to Preston Infirmary. Mr. Foster is vice-chairman of the hospital's board. The two-storeyed house, though much added to at different dates, is old, and its foundations even older. It stands in eight acres of grounds, and is named after the stream which runs through the property. This sufficiently indicates the attractions of the place, which has been occupied for the past sixteen years as the private house of Mr. Cuthbert Pyke.

### A SIMPLE POLICY FOR SHEFFIELD.

The financial condition of the Sheffield hospitals, which jointly suffer an annual deficit of £20,000, the shortage of beds which means a joint waiting-list of 1,100, and the difficulty with which £86,000 has been raised in six months in response to the recent appeal for £100,000, are discouraging facts. There has always been an apathy in Sheffield in regard to hospital questions. Only lately the plan to establish a new orthopædic hospital had to be abandoned in consequence of the meagre support it received. Even workmen's contributions on which most industrial centres in the north are prone to plume themselves are a half-hearted affair in Sheffield. We believe that the firms which encourage them can almost be counted on the fingers, and yet Sheffield is supposed to have 100,000 workers. A meeting between employers and employed should be arranged to discuss joint action, and a model for their organisation can be found in the arrangements which have been successful at Hartshill in North Staffordshire, and to the same extent at Leicester and elsewhere. Why should prosperous and wealthy Sheffield have fallen to its present wretched condition? Wake up, Sheffield! Now that the hospitals have issued a joint appeal merely to pay off arrears, the same committee should approach the employers' organisations and those of the workmen to see what can be done to secure the hospitals' maintenance. It is not a difficult matter, or a new experiment. It is merely a step to bring Sheffield out of the rut into which the city has fallen in regard to its hospitals for many years.

### WHAT HAPPENS TO PATIENTS' LEAVINGS?

At one of the provincial hospitals the other day a speaker declared that the provision bills were enormous and that complaints were made that food was wasted. In reply the Vice-Chairman said that waste was bound to occur where sick people were being fed. "Things," he said, "which patients do not eat must be sent away; others can hardly be expected to eat food that has been picked over by sick people." Food that has been "picked over" by people, ill or well, does not sound appetising; but a good cook is also an economist, and it would be interesting to compare economies. What does happen to the food which remains on patients' plates? Has no other hospital a suggestion to offer to the vice-chairman quoted above?

### AN ORGANISER OF CONCERTS.

An interesting presentation took place at the Prince of Wales' Hospital, Cardiff, when the patients and staff handed to Mr. Ernest Williams gifts of a gold-mounted fountain pen, a silver-mounted pipe, and a tobacco pouch, together with an autograph album, as recognition of the great assistance rendered by him in organising concerts at the hospital during the past two years. The men expressed a desire some months ago to give a memento to Mr. Williams, and since then small sums have been collected amongst the patients, who have also signed their names in the autograph album, together with notes of their regiments and war service. In addition to the concerts at the Prince of Wales' Hospital, Mr. Williams has also organised concerts at the other Cardiff hospitals. Over 1,230 concerts have been arranged by him since war broke out. Rejected several times by the recruiting authorities, he considered this to be a form of service which would be helpful. There is no doubt the concerts have been very beneficial to men in hospital.

### EQUAL PAY FOR MEN AND WOMEN.

The Sheppey Board of Guardians has decided on "equal pay for equal service," and has therefore fixed the salary for male and female attendants on the mental cases under its charge at eight shillings per day. So long as the same class of work is entrusted to both sexes, the men have little cause to complain; nor so far as we know, have they done so.

### IDEAS ON CO-ORDINATION IN BIRMINGHAM.

Sir David Brooks, Lord Mayor of Birmingham, presided over a public meeting, to start his scheme for the better support and co-ordination of the city hospitals. His ideas, previously expressed, favour the voluntary system, but also much closer co-operation between the different hospitals than has been the practice hitherto. He asks, for instance, why a scheme of central buying at a common centre should not be established, so that "all hospital requisites could be bought in common on better terms than the individual hospitals could obtain"? By this means he believed that much waste could be prevented, and that such appeals as were issued would produce better results. Another of his sugestions is as to various classes of patients to different hospitals, and he urged again the plan of a hospital city outside the centre, at which point accommodation for urgent cases only would be retained. Sir David has already encouraged the promotion of a hospital council or central board whose chief duty at first would be to prevent overlapping and to reduce expenditure. The prime object of the meeting was to form a scheme to relieve the Birmingham hospitals of their indebtedness, which amounts now to some £40,000 a year. The meeting pledged itself to support the appeal which is about to be issued, and the members of the Saturday Fund have already agreed to raise their weekly contributions.

### THE NOTTINGHAM WAR MEMORIAL.

The extension of Nottingham General Hospital, which was proposed as the War memorial of the county and the city, has been the subject of criticism from those who wish the War memorial to take another form. On April 28 the hospital's board resolved that, subject to the Duke of Portland's approval, the scheme should concern the General Hospital only. A later meeting has decided that the War memorial scheme should be reconsidered.

### A RETROGRADE STEP AT MANCHESTER.

By a majority of one the Manchester Board of Guardians has decided to suspend the arrangement of twenty-six years' standing, by which no case (not in need of special treatment) chargeable to the Guardians shall be sent to any institution that does not belong to the Board. The effect of this decision, unless it is reversed, will be to withdraw children from the Roman Catholic Homes and to transfer them to two large schools, said to be half empty, which belong to the Guardians. The proposal is supported merely on the ground that the Guardians' children ought to be directly under the Guardians' care. It was opposed for the same reason—namely, that the children should be removed from the workhouse atmosphere, and that they should not be herded together in the barracks which are characteristic of the Poor Law. With the latter view we agree. The present decision, decided by an odd vote, merely reverses an enlightened policy which has worked well and economically for nearly thirty years. It would be absurd to change it. Because the Guardians are the authority which is responsible for the welfare of these children it does not in the least follow that the Guardians are the right people to educate them. The Guardians are trustees and *in loco parentis*, and trustees and parents know their duty to be to choose the best schools within their means, and not to confine the children to their own houses. Since, too, there is an objection in after-life to one brought up in a Poor-Law school and its mixed company, the boarding-out system has everything in its favour.

### FINANCIAL EFFORTS FOR BECKETT HOSPITAL.

Barnsley Feast is an annual festivity lasting one week, in which an important part is played by the Charities Carnival. It helps to support the Beckett Hospital, Barnsley, which is now £9,000 in debt, but for which the miners have agreed to contribute a penny a week from their wages. It is now hoped to organise sectional subscriptions from other classes in the district, and the Hospital Saturday and Sunday Fund Committee is trying to have this arranged. Mr. H. Feasby, the Chairman of the Fund, is a keen supporter of the proposal, and in him and Mr. A. Whitham the Beckett Hospital has two old friends who will do their best to see that the various organisations for the collection of war funds do not lapse, but divert their efforts for the hospital's benefit. It is possible that the district committees attached to the Fund might extend their scope, and organise not merely local festivals, but local funds also. We hope, too, that gifts in kind from such centres will not be allowed to lapse, as they have for some unexplained reason in several other places since the armistice occurred. They are immensely useful, and the givers take perhaps more pride in them than in any monetary gift; while they can do much to keep down the high price of provisions.

### HOW CHILDREN CAN HELP.

The county of Sussex—or, to be exact, that part of the county covered by the Brighton, Hove, Central and West Sussex Sunday School Union—has furnished an excellent example of how children in health can, by a united effort, help children in sickness. In connection with the "Children's Year," the designation given by Mr. T. Vivian Rees, of Cardiff, as National President of the Sunday School Union for the year now ensuing, the branch of the Union named set out to provide and endow a cot at the Royal Alexandra Hospital for Sick Children at Brighton, the immediate objective being the sum of £525. The result was that the scholars of the schools in the district of the Union were busy all winter making articles for an exhibition and sale of work. This was held last week and realised £880, an excess of £355. With the proceeds of the "clearance sale" it is not unlikely that the net result of the effort will be £1,000. An ordinary appeal has little effect on the poor; they feel shy about giving a small contribution. But in an effort like the one under notice where they can, at a small cost, do something to help, they gladly give of their best.

### SOUTHWARK CONVALESCENT HOME SCHEME.

Southwark Board of Guardians have decided to ask the Local Government Board to approve of a scheme for the acquisition by the guardians of a suitable place as a Convalescent Home for their patients. At present they have twenty-five patients treated in a private institution at Lancing-on-Sea, whilst others are boarded out in other homes. It is felt that if the guardians had their own Convalescent Home, not only would the expense be greatly reduced, but patients being under immediate care and control, the recoveries might be expedited. A large number of phthisical cases come under the guardians' care, and it is realised that the provision for their treatment is at present inadequate.

### PHARMACISTS AND DENTAL REGISTER.

Pharmacists who have been practising as dental operators are to be treated on a different footing from other persons so acting who are not registered "dentists," if the recommendations of the Departmental Committee are adopted by the Government. Whilst the ordinary unregistered practitioner of dentistry must prove that dental work is his *chief* source of livelihood, the Committee recommend that the names of persons on the "Register of Chemists and Druggists" who can prove that their dental practice is a *substantial* one, and include the usual dental operations, be added to the Dental Register. One important proviso is made—viz., that where a registered chemist is admitted to the Dental Register he must, within the period of five years, decide which profession he intends to follow exclusively—dentistry or pharmacy. It will also be necessary for a chemist so registered to give an undertaking that he will not practise the two professions together. Failure to observe this will mean the erasure of his name from the Dental Register. It is to be noted that registered chemists who have become "dentists" in the orthodox manner after the prescribed curriculum and examination will not be affected by the Committee's proposals.

### THIS WEEK'S DRUG MARKET.

Business has been more or less at a standstill since last report appeared, for the holiday was not confined to the single statutory day. We may expect to see, however, an early resumption of activity, for the tone of the market is quite healthy. Cheaper supplies of Turkey opium have not as yet resulted in any further drop in the quotation for morphine and codeine, but lower prices are expected in the not far distant future. Menthol and camphor continue to attract attention and values are firmly maintained. Linseed and linseed oil continue to move upwards in price. Ipecacuanha and cardamoms are dearer. Among the articles which have a downward price tendency are cocaine, atropine, salol, phenazone, barbitone, calumba root, honey, thymol, and cajuput oil.

